# Biradical vs singlet oxygen photogeneration in suprofen–cholesterol systems

**DOI:** 10.3762/bjoc.12.115

**Published:** 2016-06-14

**Authors:** Fabrizio Palumbo, Francisco Bosca, Isabel Maria Morera, Inmaculada Andreu, Miguel A Miranda

**Affiliations:** 1 Instituto de Tecnología Química UPV-CSIC/ Departamento de Química, Universitat Politècnica de València, Camino de Vera s/n, 46022 Valencia, Spain; 2 Unidad Mixta de Investigación IIS La Fe-UPV, Hospital Universitari i Politècnic La Fe, Avenida de Fernando Abril Martorell 106, 46026 Valencia, Spain

**Keywords:** aryl ketones, hydrogen abstraction, lipid peroxidation, photoproducts, triplet excited state

## Abstract

Cholesterol (Ch) is an important lipidic building block and a target for oxidative degradation, which can be induced via free radicals or singlet oxygen (^1^O_2_). Suprofen (SP) is a nonsteroidal anti-inflammatory drug that contains the 2-benzoylthiophene (BZT) chromophore and has a π,π* lowest triplet excited state. In the present work, dyads (*S*)- and (*R*)-SP-α-Ch (**1** and **2**), as well as (*S*)-SP-β-Ch (**3**) have been prepared from β- or α-Ch and SP to investigate the possible competition between photogeneration of biradicals and ^1^O_2_, the key mechanistic steps in Ch photooxidation. Steady-state irradiation of **1** and **2** was performed in dichloromethane, under nitrogen, through Pyrex, using a 400 W medium pressure mercury lamp. The spectral analysis of the separated fractions revealed formation of two photoproducts **4** and **5**, respectively. By contrast, under the same conditions, **3** did not give rise to any isolable Ch-derived product. These results point to an intramolecular hydrogen abstraction in **1** and **2** from the C7 position of Ch and subsequent C–C coupling of the generated biradicals. Interestingly, **2** was significantly more photoreactive than **1** indicating a clear stereodifferentiation in the photochemical behavior. Transient absorption spectra obtained for **1**–**3** were very similar and matched that described for the SP triplet excited state (typical bands with maxima at ca. 350 nm and 600 nm). Direct kinetic analysis of the decay traces at 620 nm led to determination of triplet lifetimes that were ca. 4.1 μs for **1** and **2** and 5.8 μs for **3**. From these data, the intramolecular quenching rate constants in **1** and **2** were determined as 0.78 × 10^5^ s^−1^. The capability of dyads **1**–**3** to photosensitize the production of singlet oxygen was assessed by time-resolved near infrared emission studies in dichloromethane using perinaphthenone as standard. The quantum yields (Φ_Δ_) were 0.52 for **1** and **2** and 0.56 for **3**. In conclusion, SP-α-Ch dyads are unique in the sense that they can be used to photogenerate both biradicals and singlet oxygen, thus being able to initiate Ch oxidation from their triplet excited states following either of the two competing mechanistic pathways.

## Introduction

Among the constituents of cell membranes, cholesterol (Ch) is the most important lipidic building block. It is required for permeability, fluidity, and integrity of all animal cell membranes. However, as an unsaturated lipid, Ch is susceptible to oxidative degradation, which can result in potentially pathologic consequences encompassing from inflammation to cardiovascular and Alzheimer diseases [1–2]. This type of damage can be induced via free radicals or singlet oxygen (^1^O_2_) [3–4]. The former generally involves hydrogen abstraction (HA) of an allylic hydrogen and can be achieved by photosensitizing agents in combination with UVA light. The latter involves energy transfer from the photosensitizer triplet excited state to ground state molecular oxygen [5–6].

**Figure 1:**
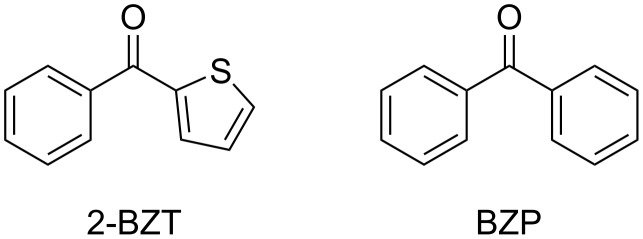
Chemical structure of the photosensitizing chromophores benzophenone (BZP) and 2-benzoylthiophene (BZT).

Ketoprofen (KP) is a nonsteroidal anti-inflammatory drug that contains the benzophenone (BZP, Figure 1) chromophore and displays a n,π triplet excited state [7–9], whereas tiaprofenic acid (TPA) is a related drug that includes the 2-benzoylthiophene (BZT, Figure 1) chromophore and has a π,π* lowest triplet excited state [9–10]. Generally, the photochemical reactivity of the n,π* triplet state is higher than that of π,π* triplet state. It is also accepted that ketones with lowest-lying π,π* triplets react predominantly via thermal population of the higher energy n,π* states.

In this context, we have previously shown that the electronic nature of the involved triplet excited state displays a marked influence on the photobehavior of ketone-Ch dyads. Hence, KP-α-Ch dyads are suitable to generate biradicals by intramolecular HA from the C7-allyl position of Ch [11–12], whereas the TPA-α-Ch analogs are unreactive via HA but they generate singlet oxygen efficiently [13].

**Figure 2:**
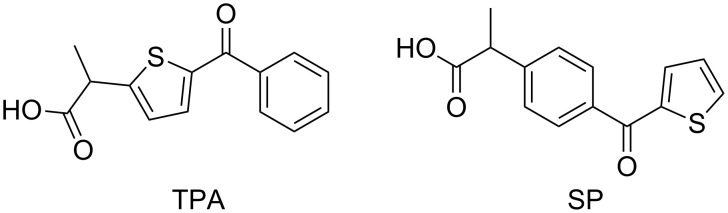
Chemical structure of tiaprofenic acid (TPA) and suprofen (SP).

Suprofen (SP) is another nonsteroidal anti-inflammatory drug, which contains a BZT chromophore. The only structural difference between SP and TPA is the site of attachment of the propionic acid side chain (Figure 2), which is the benzoyl or the thenoyl group, respectively [14–15]. Interestingly, this apparently minor modification leads to a smaller energy gap between the T_1_ (ππ*) and the T_2_ (nπ*) states in SP than in TPA (ca. 3 vs 7 kcal/mol, respectively) [16]. Therefore, HA processes could be enhanced in the SP derivatives.

**Figure 3:**
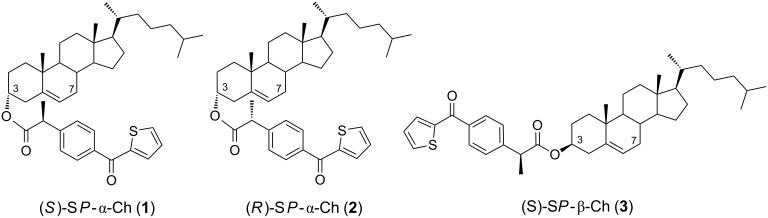
Chemical structures of dyads **1**–**3**.

With this background, dyads (*S*)- and (*R*)-SP-α-Ch (**1** and **2**), as well as (*S*)-SP-β-Ch (**3**) have been prepared in the present work from β- or α-Ch and SP (Figure 3) in order to investigate the possible competition between photogeneration of biradicals and ^1^O_2_, the key mechanistic steps in Ch photooxidation.

## Results and Discussion

### Preparation of dyads

Compounds **1**–**3** were prepared by esterification of α- or β-Ch with racemic SP following standard procedures [13]. They were purified and resolved into the pure diastereoisomers by fractional crystallization from hexane/ethyl acetate. Ester **1** was obtained as a pure solid, while its diastereoisomer **2** remained as a viscous oil after elimination of the solvent from the filtered solution. In order to make an unambiguous stereochemical assignment, authentic samples of **1** and **2** were prepared by direct esterification of (*R*)- and (*S*)-SP with α-Ch [17].

### Steady-state photolysis

**Scheme 1:**
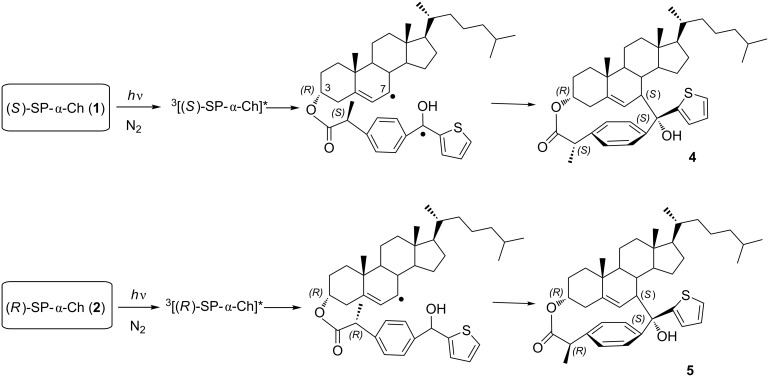
Formation of products **4** and **5** upon photolysis of dyads **1** and **2**.

In order to investigate photoproducts formation, steady-state irradiation of dichloromethane solutions (ca*.* 10^−3^ M) of **1**–**3** was performed under nitrogen, using a Pyrex filter and a 400 W medium pressure mercury lamp. The reaction progress was followed by TLC and NMR. The resulting photomixtures were submitted to silica gel column chromatography, using hexane/ethyl acetate (95:5 v/v) as eluent. The spectral analysis of the separated fractions revealed the formation of two new diastereomeric photoproducts **4** and **5** from dyads **1** and **2**, respectively (Scheme 1). By contrast, (S)-SP-β-Ch (**3**) did not give rise to any isolable Ch-derived product; this is in agreement with conformational restrictions, which do not allow an effective approach between the two active moieties. The nature of the photoproducts formed from **1** and **2** point to an intramolecular HA from the C7 position of Ch and subsequent C–C coupling of the generated biradicals.

**Figure 4:**
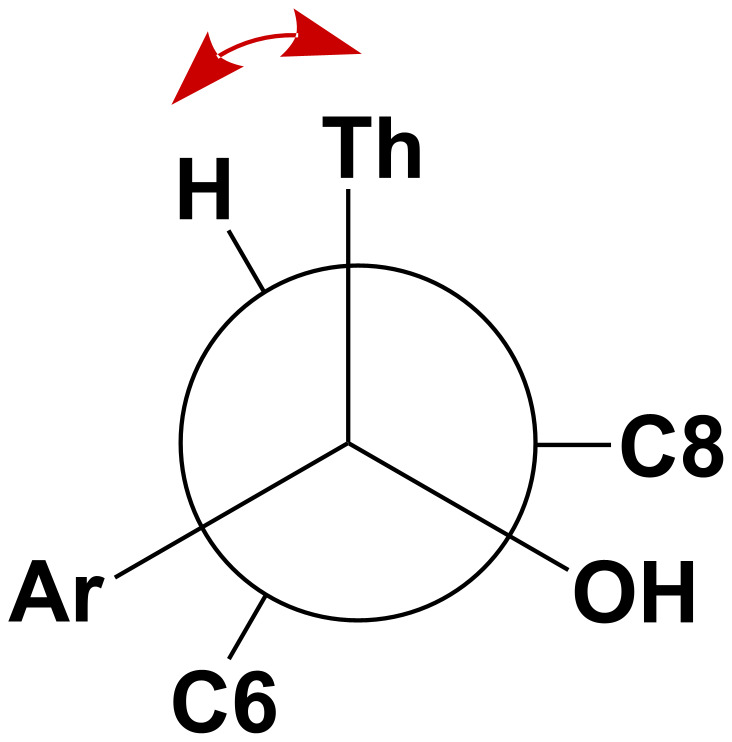
Diagnostic NOE interactions in compounds **4** and **5**.

The structures of compounds **4** and **5** were unambiguously assigned on the basis of their NMR spectroscopic data (^1^H, ^13^C, HSQC and NOEDIFF) and mass spectrometry analysis, including high-resolution measurements. Because of the rigidity of the steroidal skeleton, NOE experiments were necessary to assign the stereochemistry of the new chiral centers generated upon photocyclization. In both photoproducts, the most relevant interaction was found between the allylic proton at C7 and the protons of the thiophene ring (Figure 4). More details are provided in the Supporting Information File 1.

**Figure 5:**
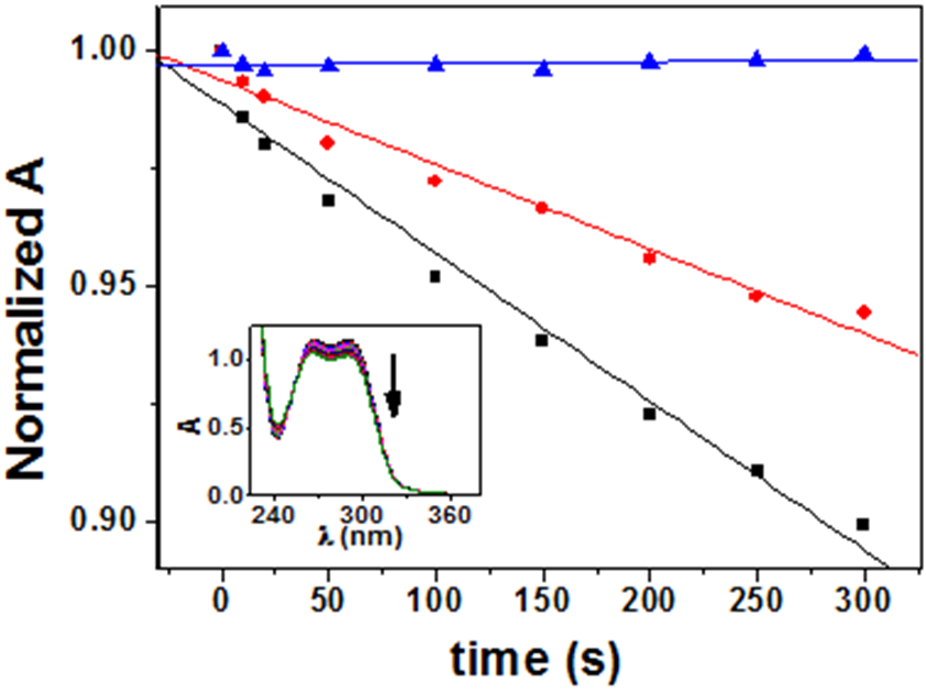
Decrease of the absorbance at 290 nm upon irradiation in CH_2_Cl_2_ under N_2_ for **1** (red circles), **2** (black squares) and **3** (blue triangles) as a function of the irradiation time. Inset: changes in the absorption spectrum of a deaerated dichloromethane solution of **1** after increasing irradiation times with monochromatic light at λ = 266 nm.

Having established the nature of the occurring photochemical reactions, it appeared interesting to explore the possible stereodifferentiation in the HA process. Thus, irradiation of **1**–**3** was performed with monochromatic light at 266 nm in CH_2_Cl_2_ (ca*.* 10^−5^ M solutions), under nitrogen. The changes were monitored by UV-spectrophotometry, following the decrease in the maximum absorption at 290 nm (inset of Figure 5), which is consistent with reduction of the BZT chromophore.

Dyads **1** and **2** were efficiently photolyzed under anaerobic conditions, whereas **3** was markedly unreactive. Interestingly, **2** was significantly more photoreactive than **1** indicating a clear stereodifferentiation in the photochemical behavior.

### Laser flash photolysis (LFP)

**Figure 6:**
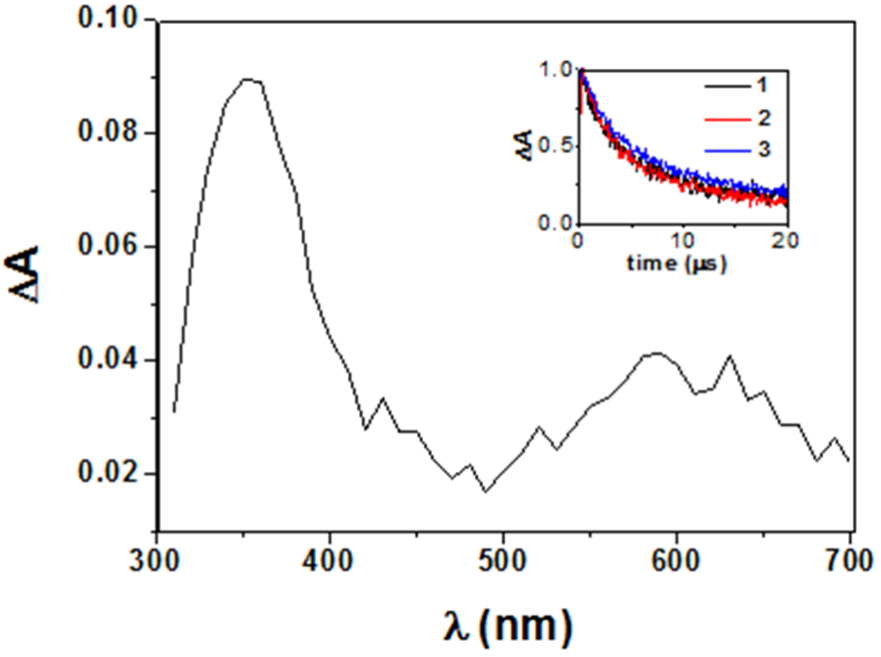
Transient absorption spectra for dyad **1** in CH_2_Cl_2_ 1 μs after laser pulse (λ_exc_ = 355 nm). Inset: Normalized decays of the triplets generated from dyads **1**–**3** monitored at 620 nm.

The studies were carried out in dichloromethane under anaerobic atmosphere at λ_exc_ = 355 nm. Transient absorption spectra acquired for **1**–**3** (Figure 6) were all very similar to that previously reported for the triplet excited state of SP, with maxima at ca. 350 nm (major) and 600 nm (minor) [18].

**Scheme 2:**
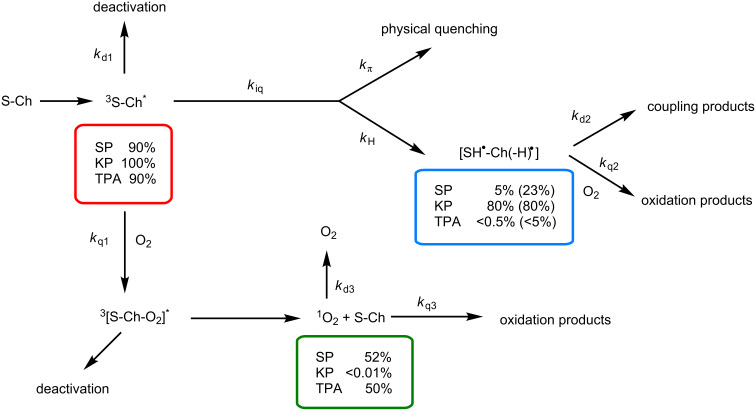
Photoreaction pathways generating biradical and singlet oxygen species of a sensitizer (S), like SP, KP or TPA, covalently linked to Ch. The obtained percentages under anaerobic conditions are given in parenthesis.

An overall mechanistic picture is shown in Scheme 2 and a summary of photophysical parameters is provided in Table 1, together with reference values from the literature [19–21]. The direct kinetic analysis of the decay traces at 620 nm (Figure 6 inset) led to determination of triplet lifetimes (τ_T_) that were ca. 4.1 μs for **1** and **2** and 5.8 μs for **3**. From these data, the intramolecular quenching rate constants were determined as *k*_iq_ = 1/τ_T(_**_1_**_ or _**_2)_** − 1/τ_T(_**_3_**_)_, and the value obtained for **1** and **2** was 0.78 × 10^5^ s^−1^.

**Table 1:** Photophysical parameters of dyads in CH_2_Cl_2_.

Parameters	(*S*)- or (*R*)-SP-α–Ch (**1**, **2**)	KP-α-Ch^a^

Φ_isc_	0.9^b^	1.0^c^
*k*_d1_ [s^−1^]	1.7 × 10^5d^	5.9 × 10^5^
*k*_iq_ [s^−1^]	7.8 × 10^4e^	1.0 × 10^8^
*k*_H_ [s^−1^]	6.2 × 10^4f^	8.0 × 10^7^
*K*_π_ [s^−1^]	1.6 × 10^4f^	2.0 × 10^7^
*k*_q1_ [M^-1^ s^−1^]	0.4 × 10^9^	0.6 × 10^9^
*Φ*_Δ_	0.52	<0.01
*k*_d2_ [s^−1^]	ND^g^	5.0 × 10^6^
*k*_q2_ [M^−1^ s^−1^]	ND^g^	3.6 × 10^9^
*k*_d3_ [s^−1^]	1.4 × 10^h^	1.3 × 10^4^
*k*_q3_ [M^−1^ s^−1^]	5.7 × 10^4i^	5.7 × 10^4i^
τ_T_ [μs]	4.10	<0.01

^a^Values taken from ref. [13]; ^b^value taken from ref. [15]; ^c^value taken from ref. [19]; ^d^*k*_d1_= 1/τ_T (_**_3_**_)_; ^e^the intramolecular quenching rate constants were estimated as *k*_iq_ = 1/τ_T(_**_1_**_ or _**_2)_** – 1/τ_T(_**_3_**_)_; ^f^the rate constants for HA (*k*_H_) and physical quenching by the π system (*k*_π_) were obtained by assuming that their ratio is similar to that determined in KP-α-Ch and that *k*_iq_ = *k*_H_ + k_π_; ^g^not determined; ^h^*k*_d3_ = 1/τ_Δ_ with perinaphthenone as photosensitizer (value taken from ref. [20]); ^i^value taken from ref. [21].

Unfortunately, in the nanosecond timescale it was not possible to detect the biradical species. Indeed, the coupling rate constant (*k*_d2_) should be similar to that of KP-α-Ch and therefore much higher than the hydrogen abstraction rate constant (*k*_H_), which is by definition lower than *k*_iq_. Consequently, biradicals are not expected to accumulate since their consumption is much faster than their formation.

It is interesting to note that the reverse is true that for the KP-α-Ch analogs, where *k*_d2_ is lower than *k*_H_ (Table 1). Therefore, biradical accumulation is indeed observed in this case, because the coupling products are generated much more slowly.

### Singlet oxygen generation

**Figure 7:**
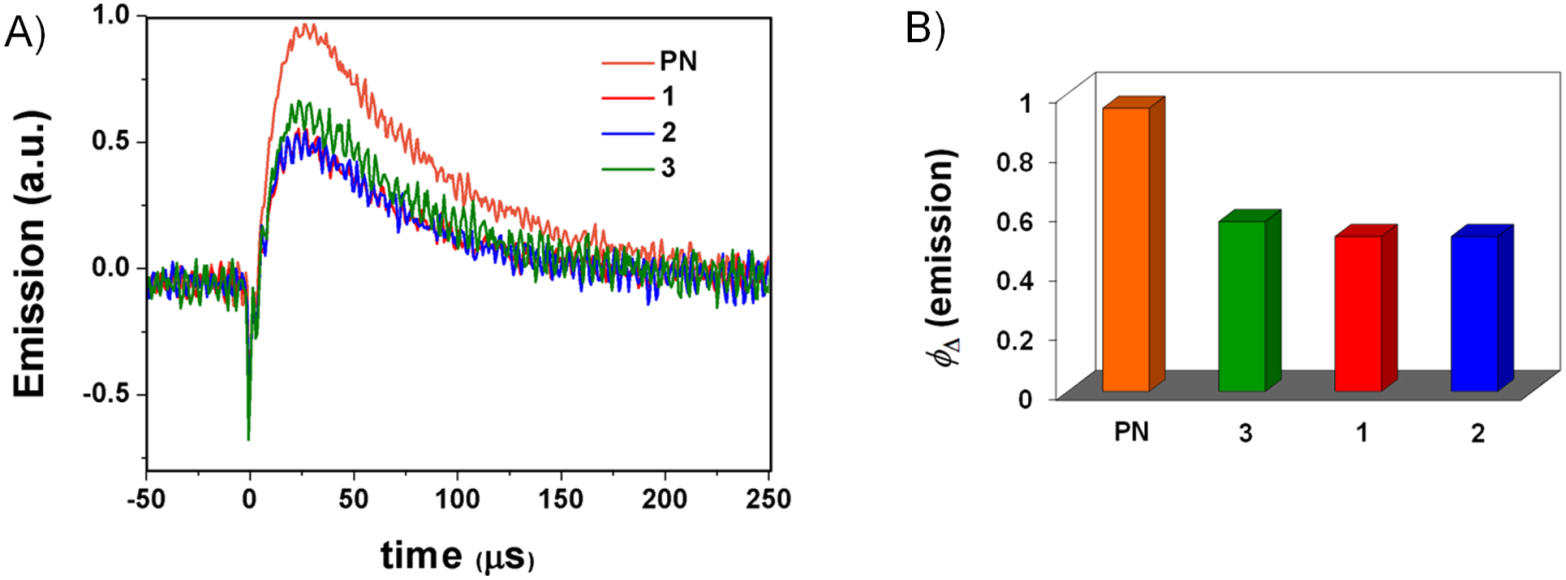
Time-resolved experiments at 1270 nm upon excitation at 308 nm of aerated CH_2_Cl_2_ solutions of **1**–**3**, using perinaphthenone as standard for comparison. A) Luminiscence decays of ^1^O_2_. B) Formation of ^1^O_2_.

To assess the capability of dyads **1**–**3** to photosensitize the production of excited singlet molecular oxygen (^1^O_2_ or ^1^Δ_g_), time-resolved near infrared emission studies were carried out in dichloromethane using perinaphthenone (PN) as standard. The formation of this reactive oxygen species was detected by its luminescence at 1270 nm, using a germanium diode as detector. The singlet oxygen lifetime (Figure 7A) was found to be ca. 70 μs in all cases (in agreement with the^1^O_2_ lifetime reported in the literature [20] for the same solvent). The photosensitized singlet oxygen production was established with a quantum yield (Φ_Δ_) of 0.52 for **1** and **2** and 0.56 for **3** (Table 1 and Figure 7B).

### Biradical vs singlet oxygen

Although it was not possible to detect the biradical species by means of LFP in SP-α-Ch systems, its generation (5%) has been chemically proven by obtaining the coupling products **4** and **5**. Moreover, the triplet excited states of **1** and **2** were quenched by O_2_ to generate ^1^O_2_ efficiently (52%). Interestingly, the TPA analogs (also with π,π* character) are unreactive via intramolecular HA (<0.5%), while they produce ^1^O_2_ with a Φ_Δ_ = 0.5. By contrast, for KP derivatives (^3^n,π*), efficient photogeneration of 7-allyl-Ch biradicals (80%) is observed, and ^1^O_2_ production is negligible (<0.01 ).

In conclusion, SP-α-Ch dyads are unique in the sense that they can be used to photogenerate both biradicals and singlet oxygen, thus being able to initiate Ch oxidation from their triplet excited states following either of the two competing mechanistic pathways.

## Experimental

### General

Suprofen and β-cholesterol were commercially available. Solvents and other reagents were used as received from the supplier without additional purification. ^1^H NMR and ^13^C NMR spectra were recorded in CDCl_3_ as solvent on a Bruker AC-300 at 300 and 75 MHz, respectively, and the NMR chemical shifts are reported in ppm downfield from an internal solvent peak. Ultraviolet absorption spectra were recorded on a Varian Cary 300 scan UV–vis spectrophotometer. All reactions were monitored by analytical TLC with silica gel 60 F_254_ revealed with ammonium molybdate reagent. The residues were purified through silica gel 60 (0.063–0.2 mm). Exact mass was obtained by Waters ACQUITY™ XevoQToF spectrometer.

### Laser flash photolysis (LFP) measurements

As described in [17], a pulsed Nd:YAG laser was used for the excitation at 355 nm. The single pulses were of ~10 ns duration, and the energy was from 10 to 1 mJ/pulse. The LFP system consisted of the pulsed laser, the Xe lamp, a monochromator and a photomultiplier made up of a tube, housing and power supply. The output signal from the oscilloscope was transferred to a personal computer. All experiments were performed at room temperature. The samples were dissolved in dichloromethane to have an absorbance of ca*.* 0.30 at 355 nm, and solutions were deareated by bubbling nitrogen.

### Steady-state photolysis of dyads **1** and **2**

Solutions of **1**, **2** or **3** (ca. 10^−4^ M concentration) were irradiated, under anaerobic conditions, with monochromatic light at 266 nm using the Xe lamp of a Photon, Tecnology spectrofluorometer equipped with monochromator. The changes were monitored by UV–vis spectrophotometry following the decrease in the absorption at 290 nm.

To preparative scale, deaerated dichlorometane (20 mL) solutions of (*S*)- or (*R*)-α-Ch dyads **1** and **2** (150 mg, 0.24 mmol) were irradiated for 8 h through Pyrex with a 400 W medium pressure mercury lamp. After this time the reaction mixtures were concentrated under reduced pressure, and the photomixtures were submitted to silica gel column chromatography, using hexane/ethyl acetate (eluent: 98:2), which afforded the pure photoproducts **4** and **5**.

### Data for compounds **4** and **5**

#### Photoproduct **4** (51%)

^1^H NMR (CDCl_3_, 300 MHz) δ 0.53 (s, 3H), 0.79 (d, *J* = 6.6 Hz, 3H), 0.80 (d, *J* = 6.6 Hz, 3H), 0.82 (s, 3H), 0.87 (d, *J* = 6.6 Hz, 3H), 1.57 (d, *J* = 7.2 Hz, 3H), 0.90–1.98 (complex signal, 24H), 2.18 (m, 1H), 2.49 (m, 1H), 2.69 (s, 1H), 3.09 (m, 1H), 3.62 (q, *J* = 7.2 Hz, 1H), 4.59 (m, 1H), 4.70 (m, 1H), 6.44 (dd, *J* = 8.1 Hz, 2.0 Hz, 1H), 6.83 (dd, *J* = 8.1 Hz, 2.0 Hz, 1H), 6.99 (dd, *J* = 5.1 Hz, 3.6 Hz, 1H), 7.11 (dd, *J* = 3.6 Hz, 1.2 Hz, 1H), 7.25 (dd, *J* = 5.1 Hz, 1.2 Hz, 1H), 7.28 (m, 1H), 7.74 (dd, *J* = 8.1 Hz, 2.0 Hz, 1H); ^13^C NMR (CDCl_3_, 75 MHz) δ 10.2, 13.6, 18.8, 18.9, 20.5, 22.6, 22.9, 23.8, 25.5, 26.7, 28.1, 33.1, 35.8, 36.1, 36.2, 38.1, 39.2, 39.6, 40.2, 41.1, 42.9, 46.6, 46.7, 50.2, 56.1, 70.2, 83.9, 124.7, 124.9, 125.0, 125.9, 126.1, 126.7, 128.0, 135.7, 139.7, 146.8, 156.2, 173.4; HRMS–EI (*m*/*z*): [M – H]^+^ calcd for C_41_H_55_O_3_S, 627.3866; found, 627.3865

#### Photoproduct **5** (53%)

^1^H NMR (CDCl_3_, 300 MHz) δ 0.53 (s, 3H), 0.79 (d, *J* = 6.6 Hz, 3H), 0.80 (d, *J* = 6.6 Hz, 3H), 0.82 (s, 3H), 0.87 (d, *J* = 6.6 Hz, 3H), 1.49 (d, *J* = 7.2 Hz, 3H), 0.90–1.97 (complex signal, 24H), 2.14 (m, 1H), 2.46 (m, 1H), 2.70 (s, 1H), 3.06 (m, 1H), 3.57 (q, *J* = 7.2 Hz, 1H), 4.55 (dd, *J* = 5.4 Hz, 1.5 Hz, 1H), 4.68 (m, 1H), 6.48 (dd, *J* = 8.1 Hz, 2.0 Hz, 1H), 6.92 (dd, *J* = 8.1 Hz, 2.0 Hz, 1H), 6.98 (dd, *J* = 5.1 Hz, 3.6 Hz, 1H), 7.09 (dd, *J* = 3.6 Hz, 1.2 Hz, 1H), 7.19 (dd, *J* = 8.1 Hz, 2.0 Hz, 1H), 7.24 (dd, *J* = 5.1 Hz, 1.2 Hz, 1H), 7.66 (dd, *J* = 8.1 Hz, 2.0 Hz, 1H); ^13^C NMR (CDCl_3_, 75 MHz) δ 10.2, 13.1, 18.8, 19.1, 20.5, 22.6, 22.9, 23.7, 25.9, 26.6, 28.1, 32.7, 35.3, 35.8, 36.2, 38.0, 39.2, 39.6, 40.1, 41.2, 42.9, 45.8, 46.4, 50.2, 56.1, 69.7, 84.0, 123.1, 124.7, 124.9, 125.7, 125.9, 126.7, 128.5, 130.8, 134.9, 140.1, 147.0, 156.0, 172.9; HRMS–EI (*m*/*z*): [M – H]^+^ calcd for C_41_H_55_O_3_S, 627.3866; found, 627.3846.

### Singlet oxygen measurements

As described in [13], the luminescence (1270 nm) from singlet oxygen was detected by means of an Oriel 71614 germanium photodiode (5 mm^2^) coupled to the laser photolysis cell in right-angle geometry. An excimer laser (LEXTRA50 Lambda Physik) was used for the excitation at 308 nm (laser excitation at 5 low-pulse energies for each molecule). A 5 mm thick (5 cm in diameter) 1050 nm cut-off silicon filter and a 1270 nm interference filter were placed between the diode and the cell. The photodiode output current was amplified and fed into a TDS-640A Tektronix oscilloscope via a Co-linear 150 MHz, 20 dB amplifier. The output signal from the oscilloscope was transferred to a personal computer for study. Thus, the singlet oxygen quantum yield (Φ_Δ_) of the dyads was determined in dichloromethane solutions using the same absorbance value (0.30) at 308 nm for each compound. A singlet oxygen quantum yield (Φ_Δ_) of 0.95 for perinaphthenone in dichloromethane was used as standard [22].

## Supporting Information

File 1 Copies of ^1^H, ^13^C, DEPT, HSQC and NOEDIFF spectra for photoproducts **4** and **5**.
