# Supplementary material for: Biradical vs singlet oxygen photogeneration in suprofen–cholesterol systems
Source: Beilstein J Org Chem. 2016 Jun 14;12:1196–202. doi: 10.3762/bjoc.12.115 (PMC4979683; doi:10.3762/bjoc.12.115)

Supporting Information

for

Biradical *vs* singlet oxygen photogeneration in suprofen–cholesterol systems

Fabrizio Palumbo[1], Francisco Bosca[1], Isabel M. Morera[1], Inmaculada Andreu*[2] and Miguel A. Miranda*[1]

Address: [1]Instituto de Tecnología Química UPV-CSIC/ Departamento de Química, Universitat Politècnica de València, Camino de Vera s/n, 46022 Valencia, Spain and [2]Unidad Mixta de Investigación IIS La Fe-UPV, Hospital Universitari i Politècnic La Fe, Avenida de Fernando Abril Martorell 106, 46026 Valencia, Spain

Email: Inmaculada Andreu - iandreur@qim.upv.es; Miguel A. Miranda - mmiranda@qim.upv.es

*Corresponding author

Copies of ^{1}H, ^{13}C, DEPT, HSQC and NOEDIFF spectra for photoproducts 4 and 5

Table of contents:

1. ^{1}H and ^{13}C NMR spectra of **4** S2
2. DEPT and HSQC spectra of **4** S3
3. NOEDIFF of **4** S4
4. ^{1}H and ^{13}C NMR spectra of **5** S5
5. DEPT and HSQC spectra of **5** S6
6. NOEDIFF of **5** S7

(^{1}H NMR, $CDCl_3$, 300 MHz)

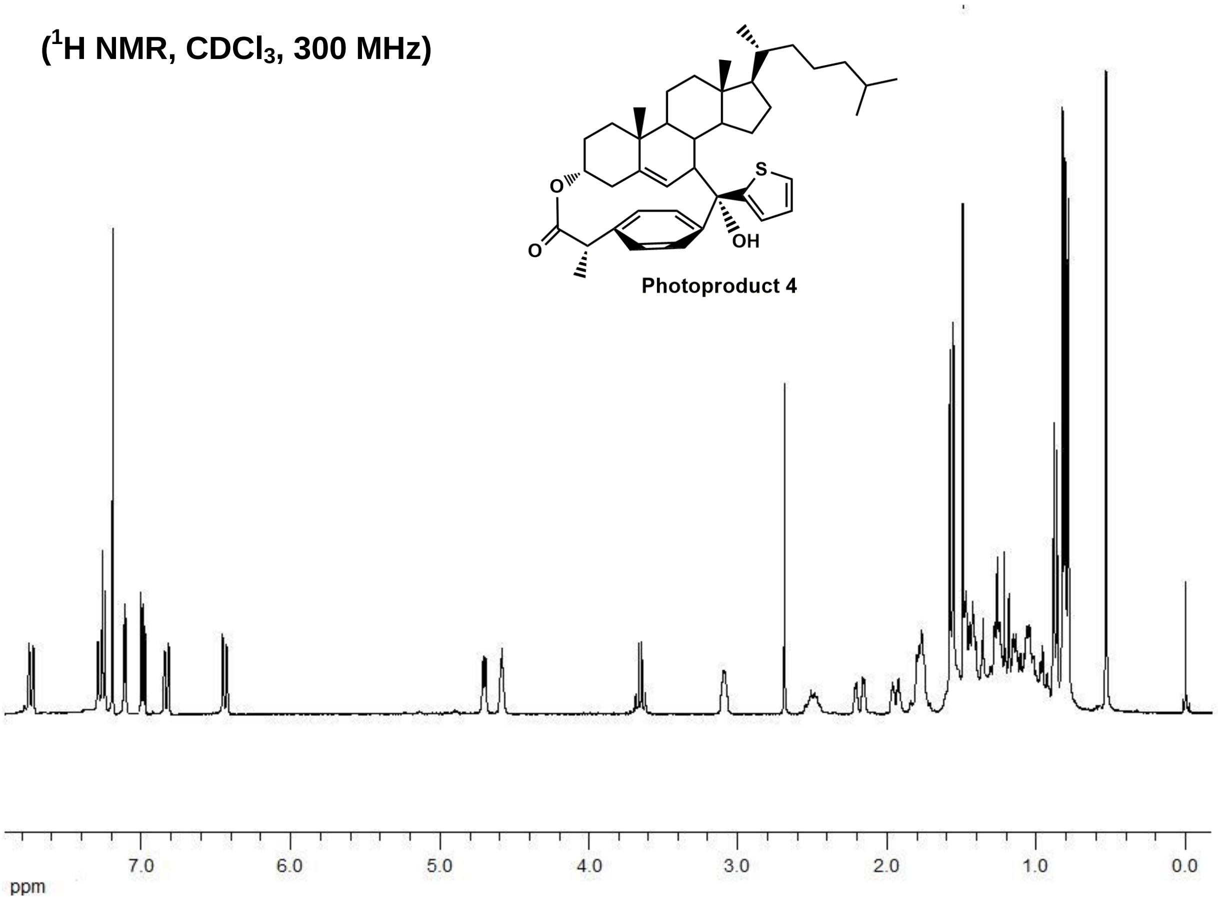

(^{13}C NMR, $CDCl_3$, 75 MHz)

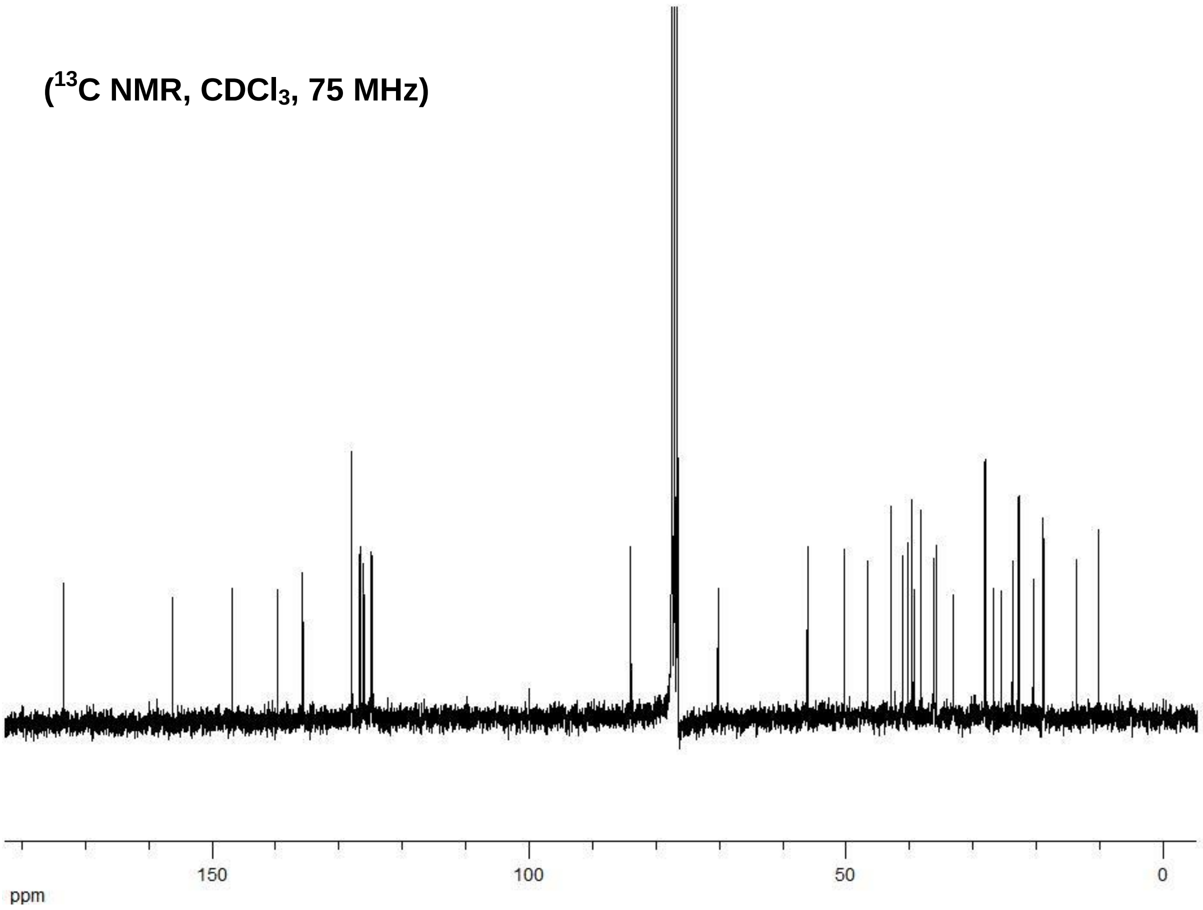

(DEPT, $CDCl_3$, 75 MHz)

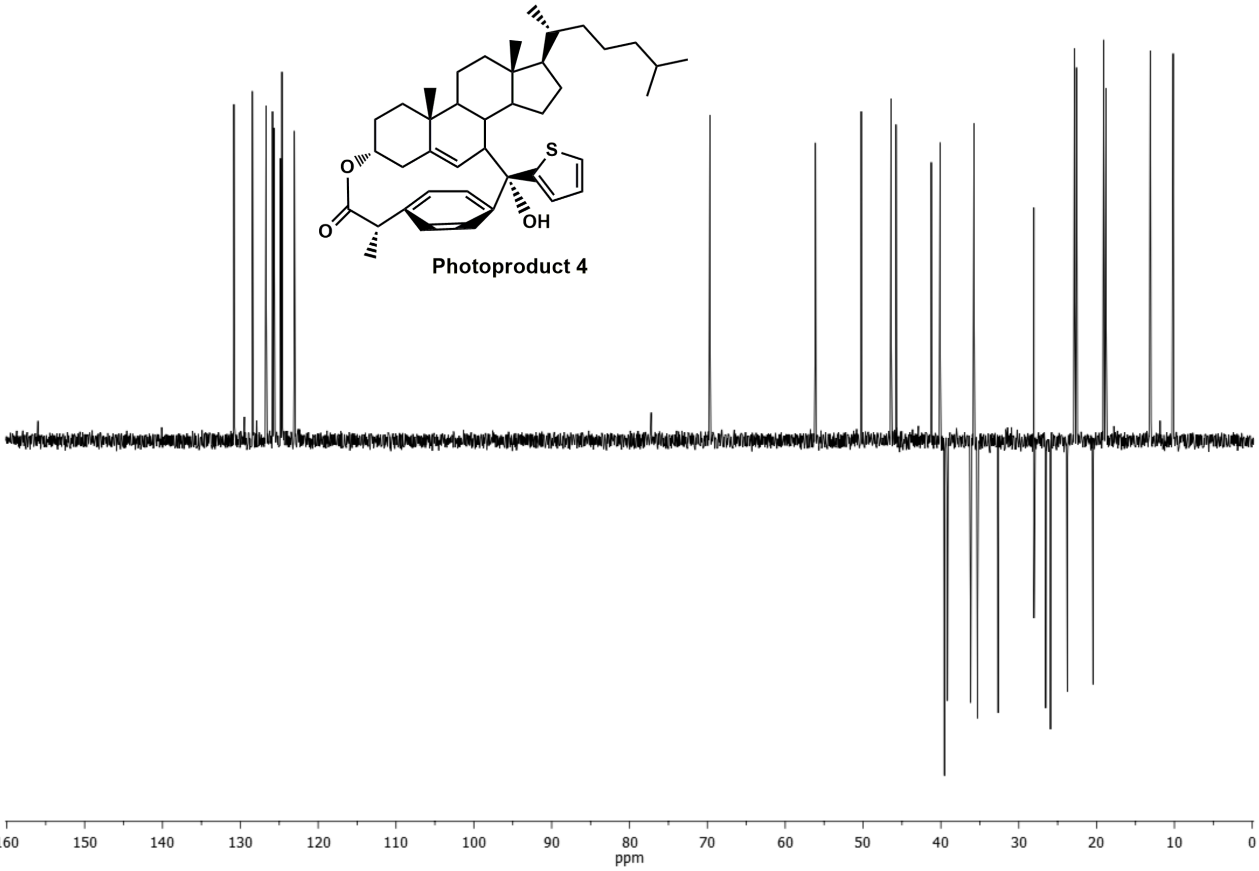

(HSQC, $CDCl_3$)

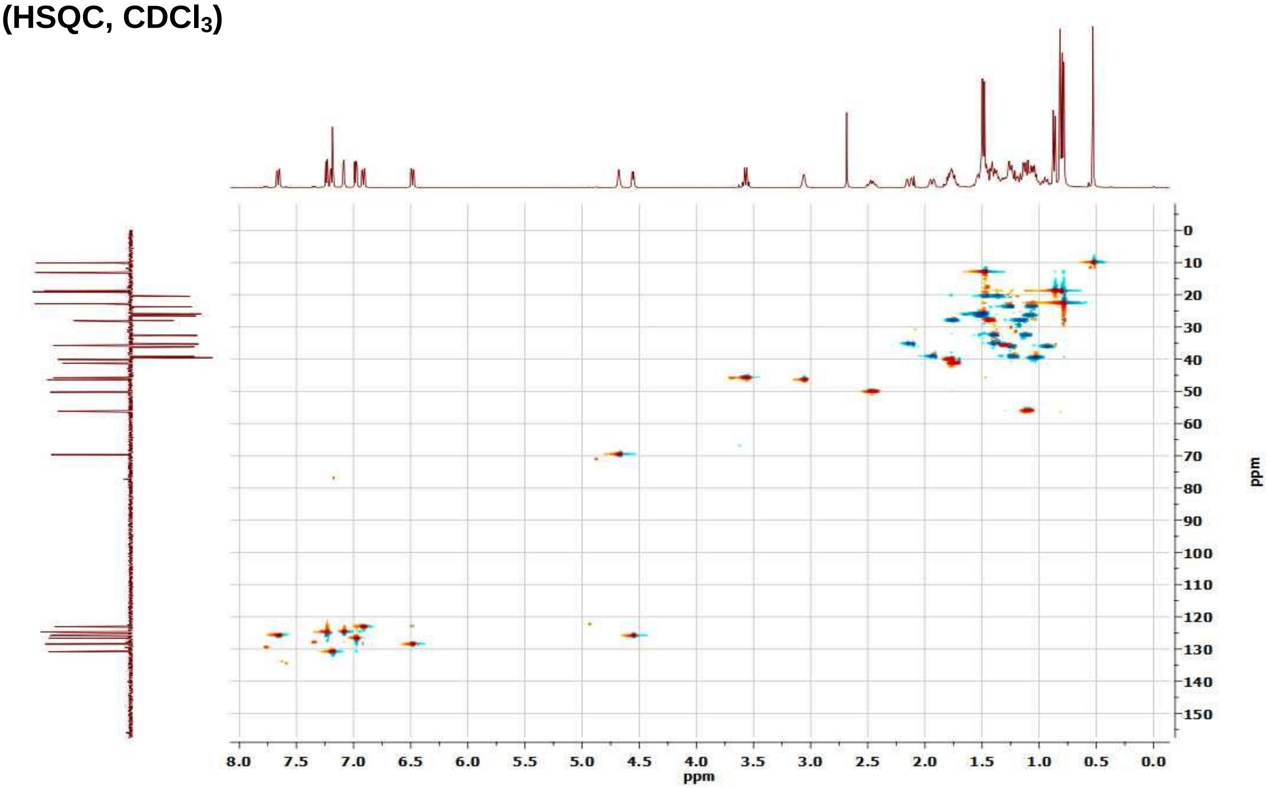

(NOEDIFF, $CDCl_3$)

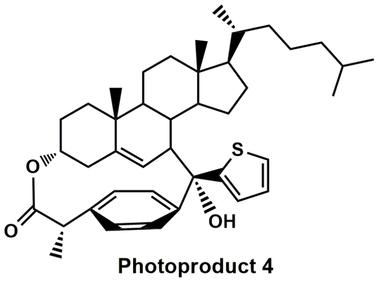

Photoproduct 4

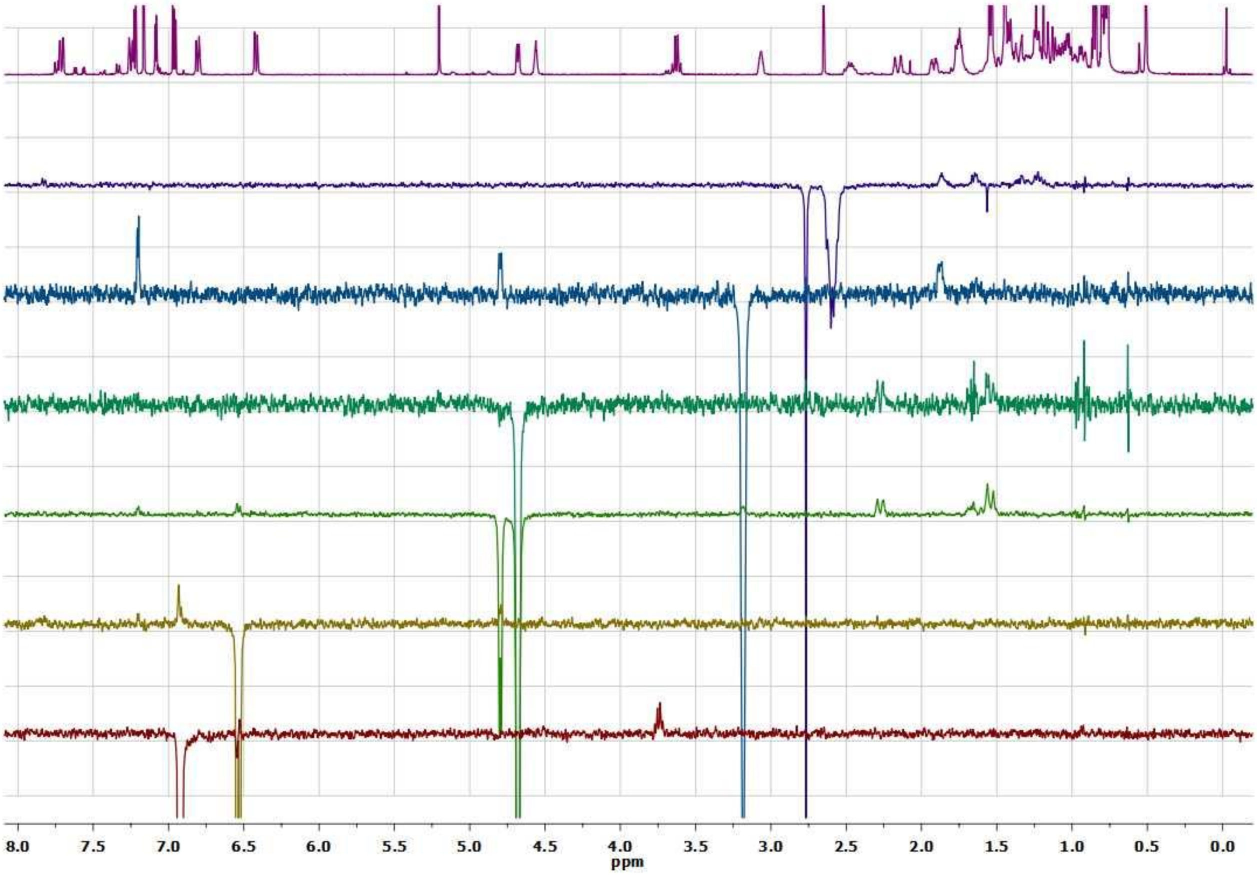

(^{1}H NMR, $CDCl_3$, 300 MHz)

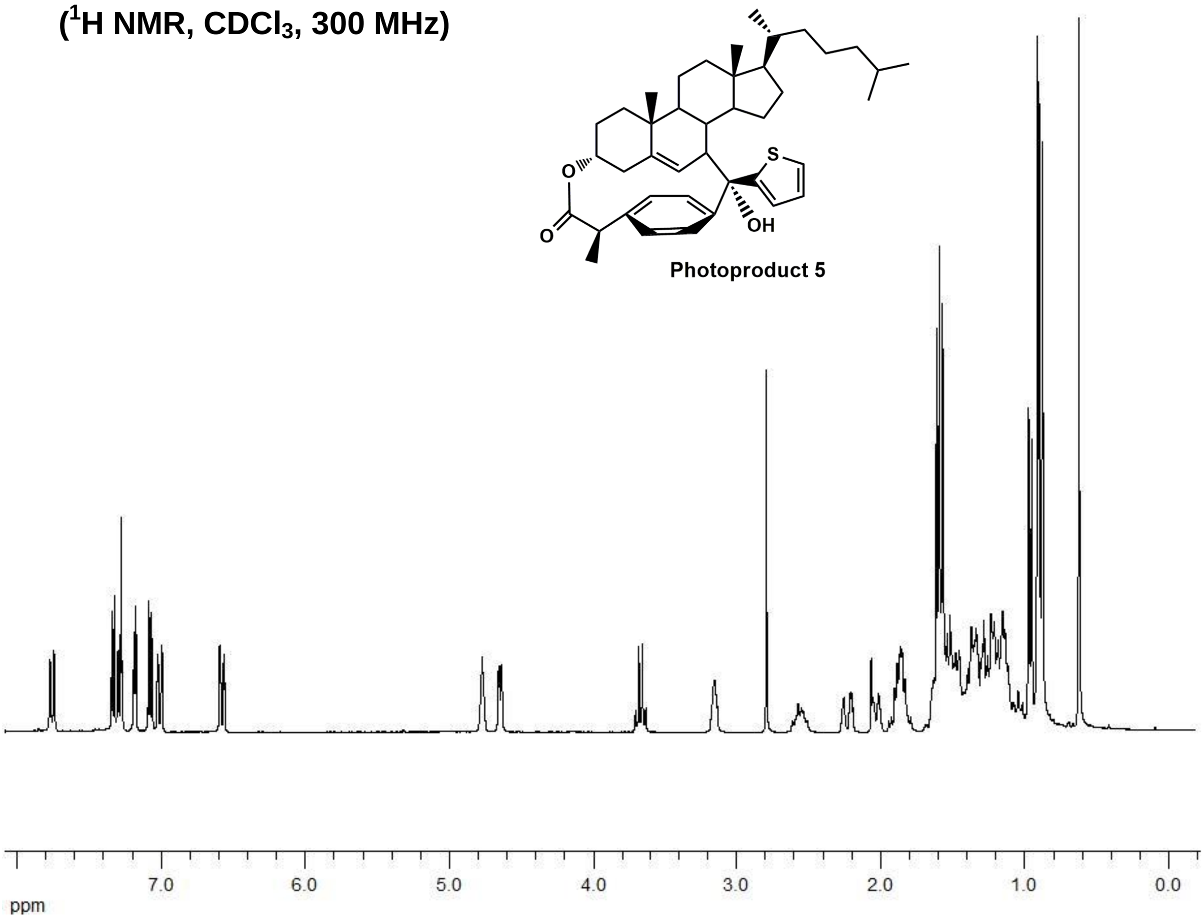

(^{13}C NMR, $CDCl_3$, 75 MHz)

($CDCl_3$, 75 MHz)

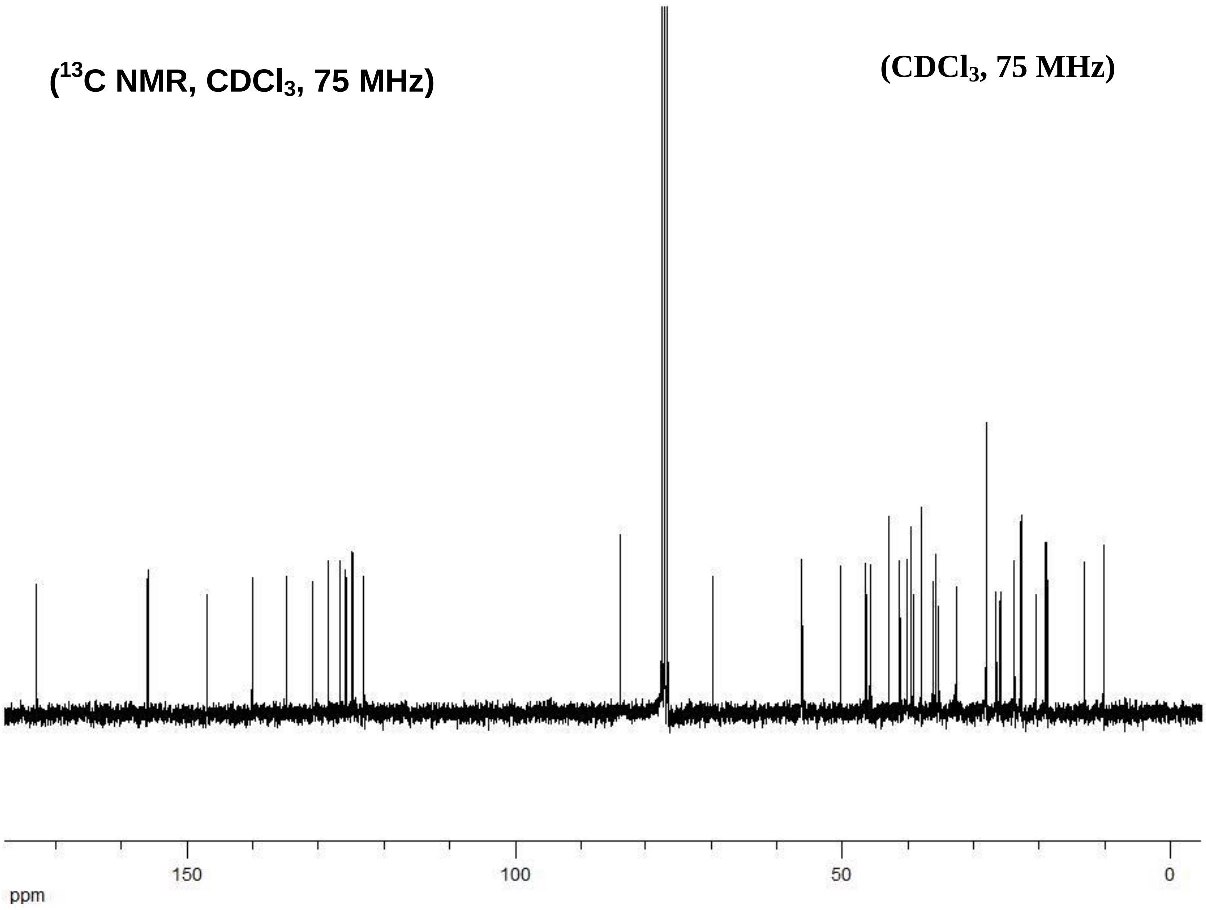

(DEPT, $CDCl_3$, 75 MHz)

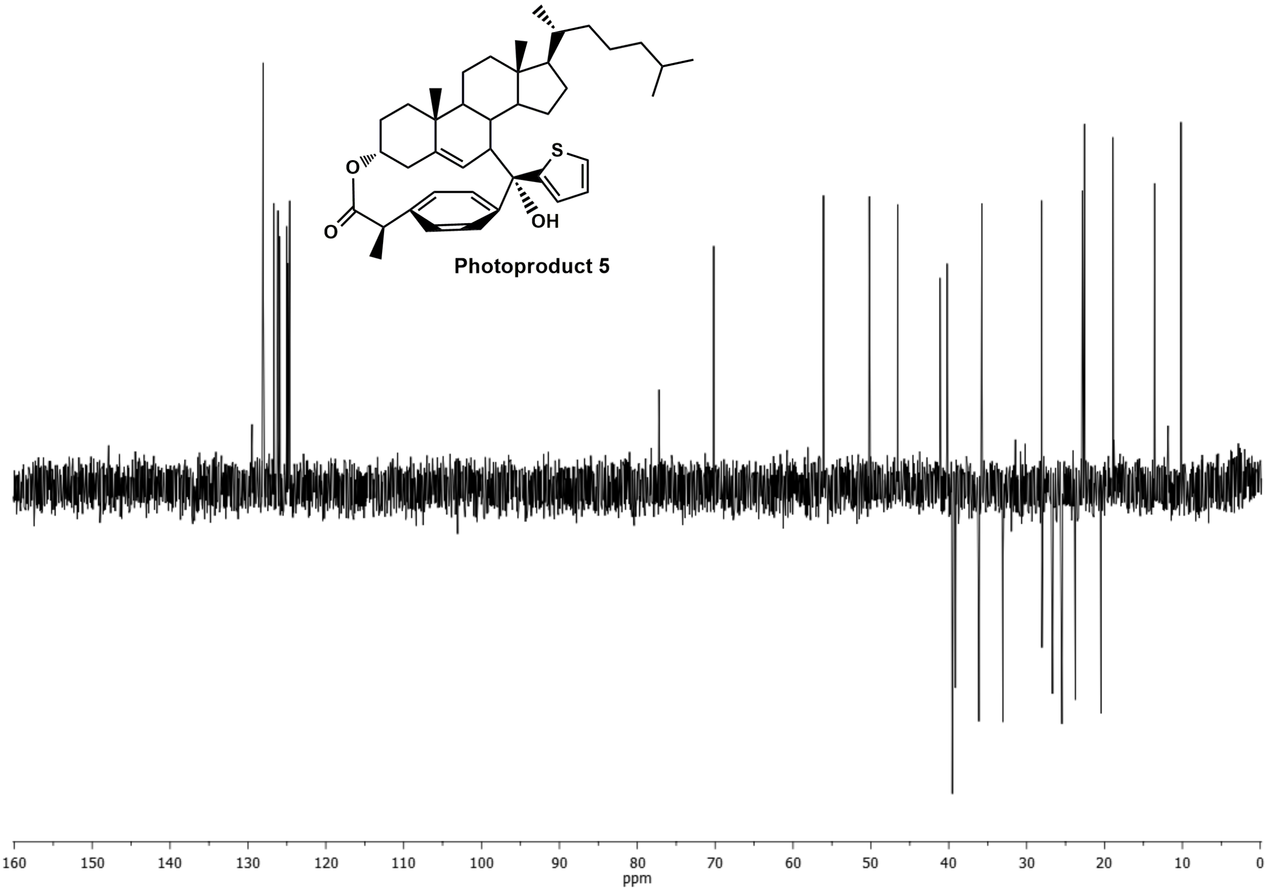

(HSQC, $CDCl_3$)

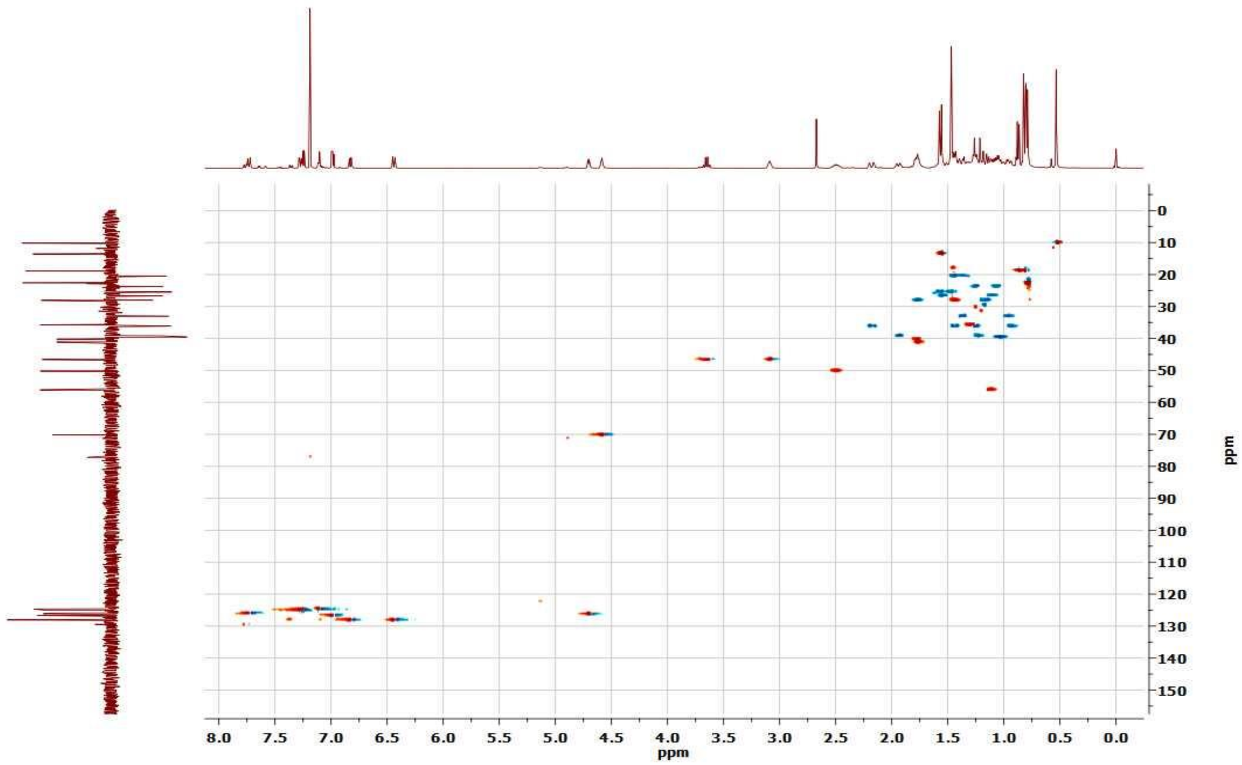

(NOEDIFF, $CDCl_3$)

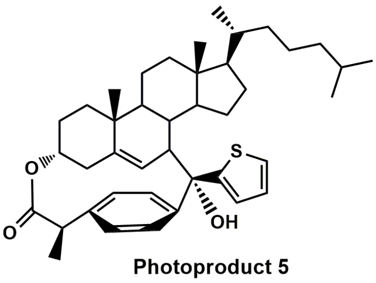

Photoproduct 5

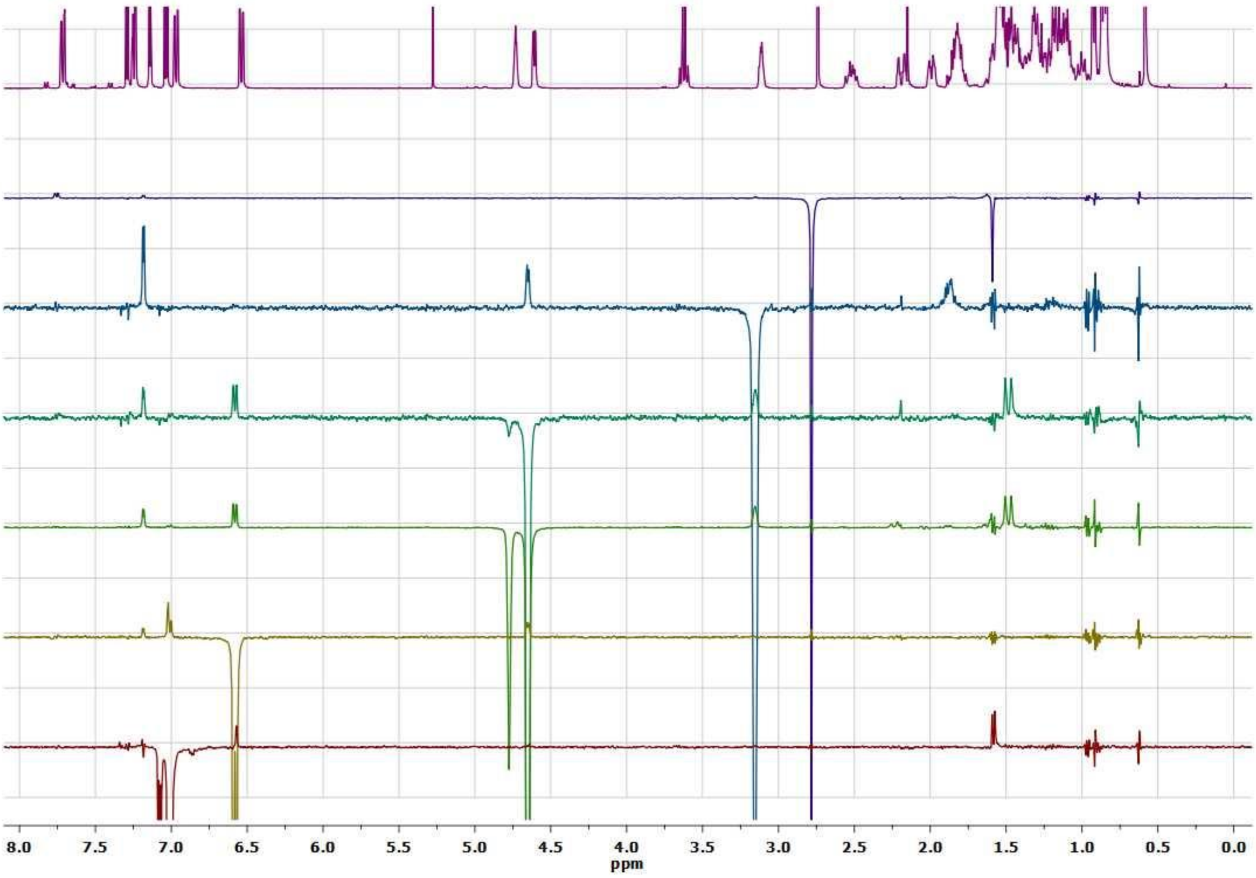

Supplement: File 1 — Copies of 1H, 13C, DEPT, HSQC and NOEDIFF spectra for photoproducts 4 and 5. [file Beilstein_J_Org_Chem-12-1196-s001.pdf]
